# Newborn Metabolic Profile Associated with Hyperbilirubinemia With and Without Kernicterus

**DOI:** 10.1111/cts.12590

**Published:** 2018-10-28

**Authors:** Molly E. McCarthy, Scott P. Oltman, Rebecca J. Baer, Kelli K. Ryckman, Elizabeth E. Rogers, Martina A. Steurer‐Muller, John S. Witte, Laura L. Jelliffe‐Pawlowski

**Affiliations:** ^1^ Department of Epidemiology and Biostatistics Global Health Sciences and the Preterm Birth Initiative University of California San Francisco San Francisco California USA; ^2^ Department of Public Health Brown University Providence Rhode Island USA; ^3^ Department of Epidemiology and Biostatistics and the California Preterm Birth Initiative University of California San Francisco San Francisco California USA; ^4^ California Preterm Birth Initiative University of California San Francisco San Francisco California USA; ^5^ Department of Pediatrics University of California San Diego La Jolla California USA; ^6^ Departments of Epidemiology and Pediatrics University of Iowa Iowa City Iowa USA; ^7^ Department of Pediatrics and the California Preterm Birth Initiative University of California San Francisco San Francisco California USA; ^8^ Department of Epidemiology and Biostatistics, Pediatrics and the California Preterm Birth Initiative University of California San Francisco San Francisco California USA; ^9^ Institute for Human Genetics University of California San Francisco San Francisco California USA; ^10^ Department of Epidemiology and Biostatistics and the California Preterm Birth Initiative University of California San Francisco San Francisco California USA

## Abstract

Our objective was to assess the relationship between hyperbilirubinemia with and without kernicterus and metabolic profile at newborn screening. Included were 1,693,658 infants divided into a training or testing subset in a ratio of 3:1. Forty‐two metabolites were analyzed using logistic regression (odds ratios (ORs), area under the receiver operating characteristic curve (AUC), 95% confidence intervals (CIs)). Several metabolite patterns remained consistent across gestational age groups for hyperbilirubinemia without kernicterus. Thyroid stimulating hormone (TSH) and C‐18:2 were decreased, whereas tyrosine and C‐3 were increased in infants across groupings. Increased C‐3 was also observed for kernicterus (OR: 3.17; 95% CI: 1.18–8.53). Thirty‐one metabolites were associated with hyperbilirubinemia without kernicterus in the training set. Phenylalanine (OR: 1.91; 95% CI: 1.85–1.97), ornithine (OR: 0.76; 95% 0.74–0.77), and isoleucine + leucine (OR: 0.63; 95% CI: 0.61–0.65) were the most strongly associated. This study showed that newborn metabolic function is associated with hyperbilirubinemia with and without kernicterus.

**Study Highlights**

**WHAT IS THE CURRENT KNOWLEDGE ON THE TOPIC?**
 A variety of neonatal and maternal characteristics are known to be associated with increased risk of hyperbilirubinemia. Hyperbilirubinemia is known to be associated with several metabolic disorders.
**what question did this study address?**
 This study sought to address whether newborn metabolic profile is associated with hyperbilirubinemia with and without kernicterus.
**WHAT DOES THIS STUDY ADD TO OUR KNOWLEDGE?**
 Forty‐one infant metabolites collected at newborn screening were shown to be associated with neonatal hyperbilirubinemia with and without kernicterus.
**HOW MIGHT THIS CHANGE CLINICAL PHARMACOLOGY OR TRANSLATIONAL SCIENCE?**
 Metabolic profile may, therefore, be important to consider when investigating etiology and risk and in identifying new targets for treatment.

Hyperbilirubinemia (jaundice) is one of the most common conditions in the first weeks of life, occurring in 60% of term infants and 80% of preterm infants.^1^ Although some degree of hyperbilirubinemia is often benign in newborns, significant unconjugated hyperbilirubinemia may have adverse consequences. Unconjugated bilirubin is difficult to excrete and can be neurotoxic at high levels.^2^

Severe unconjugated hyperbilirubinemia can damage the basal ganglia and deep grey nuclei, resulting in chronic bilirubin encephalopathy (also called “kernicterus”) and sometimes death.^2^, ^3^ Advances in hyperbilirubinemia screening and treatment have led to dramatic decreases in kernicterus rates in the United States and other industrialized nations,^4^ but the global burden is far from eliminated. More than 481,000 term or near‐term babies develop severe hyperbilirubinemia worldwide and 114,000 succumb to it each year, with 75% of cases occurring in Sub‐Saharan Africa and Southeast Asia.^4^

It is well established that preterm infants are more likely to develop hyperbilirubinemia due to an immature gastrointestinal tract and hepatic conjugating system.^2^, ^5^ Preterm infants are also more susceptible to bilirubin toxicity because of an immature blood brain barrier and clinical instability.^6^, ^7^, ^8^ Phototherapy is an effective treatment for hyperbilirubinemia, and guidelines for phototherapy in preterm infants are based on total serum bilirubin (TSB) levels.^2^, ^3^, ^4^, ^5^, ^9^, ^10^ However, the risk of bilirubin encephalopathy is not directly associated with TSB levels; kernicterus can occur in premature infants with only modestly elevated TSB levels.^5^

In addition to gestational age, there are several other known clinical risk factors, including Asian race, previous sibling with hyperbilirubinemia, and exclusive breastfeeding.^2^, ^9^ However, predicting who will eventually develop significant hyperbilirubinemia remains difficult.^11^ A number of investigators have explored creation of screening tools that utilize characteristics to identify high‐risk infants. Existing predictive models are based upon characteristics, such as TSB levels, mode of delivery, gestational age (GA), and body mass loss.^12^, ^13^, ^14^

Derangements of the metabolism are known to be a major cause of decreased bilirubin clearance.^2^ As such, we suspect that metabolic function itself may be associated with hyperbilirubinemia.

Here, we explore whether 42 metabolites collected as part of routine newborn screening (NBS) in California are associated with hyperbilirubinemia occurring with and without kernicterus and whether patterns may aid in identification of infants at increased risk of hyperbilirubinemia and new targets for treatment.

## Methods

We performed a population‐based retrospective cohort analysis utilizing data collected by the California Office of Statewide Health Planning and Development (OSHPD) for California livebirths between 2007 and 2011. The OSHPD database contains information about infant and maternal characteristics, and was combined with NBS data using date of birth, hospital of birth, birth weight, and birth time to obtain metabolic marker information. The California Department of Public Health screens all newborn infants for inborn metabolic diseases by measuring markers in a heel‐stick blood spot taken between 12 hours and 8 days after birth.^15^

After linkage with OSHPD data, there were 1,826,145 infants eligible for analysis. Infants were excluded if they had birthweights outside four standard deviations from the mean for GA by sex,^16^ GA outside the range of 22–41 weeks at birth, lacked full metabolic data, or were discharged after more than 7 days. After these exclusions, there were 1,693,658 infants included in the analysis. Exposed infants were any for whom the *International Classification of Diseases 9th revision (ICD‐9)* diagnostic code for hyperbilirubinemia (774.0–774.9) appeared in hospital discharge records during the first year of life. We randomly divided the final cohort into a training subset used to build the multivariable models of 1,270,244 infants (75% of total) and a testing subset used to verify the multivariable models of 423,414 infants (25% of total).

Infant birthweight, GA, sex, age at NBS, feeding type at the time of newborn screening (breast milk only, formula only, and mixed breast and formula), timing of diagnosis (before and after discharge for birth), and maternal race/ethnicity, education, MediCal (California's insurance for low‐income persons – a proxy for socioeconomic status), hypertension, diabetes, obesity, infection during pregnancy, and smoking during pregnancy were analyzed along with metabolites to determine their association with neonatal hyperbilirubinemia with and without kernicterus (**Table** 1). Characteristics and metabolites were chosen based on known or suspected associations with neonatal hyperbilirubinemia.

Amino acids and carnitines were measured using standardized mass spectrometry. Fluorometric enzyme assay was used to measure galactose‐1‐phosphate uridyl transferase and high‐performance liquid chromatography was used to measure thyroid stimulating hormone (TSH) and 17‐hydroxyprogesterone.^17^ All metabolites were measured in units of μmol/L (see **Table** 2 for a complete list of metabolites measured and included in analyses).

### Statistical analyses

All metabolites underwent natural log transformation prior to analysis to minimize skewness and the influence of outliers, and were treated as continuous variables. We performed χ^2^ comparisons, Student's *t* tests, and univariate logistic regression to obtain odds ratios (ORs) and 95% confidence intervals (CIs) comparing infant/maternal characteristics and metabolic markers in cases and controls. All tests were two‐sided and the threshold for significance was set at a Bonferroni‐corrected *P* value of *P* = 0.0012 (0.05/42). Backward stepwise multivariable logistic regression was used to determine which variables were associated with hyperbilirubinemia with and without kernicterus after adjustment for other factors. For hyperbilirubinemia without kernicterus modeling, all characteristics and metabolites that met Bonferroni significance in the univariate tests were considered for inclusion and removed from the model at *P* > 0.05. Given small sample size, for infants with kernicterus, variables were considered for inclusion at *P* < 0.10 and removed from the model at *P* > 0.05. Due to the well‐documented tendency of feeding type and age at NBS to influence metabolite levels,^18^, ^19^ we adjusted for these factors in all models. Multicollinearity among predictors was assessed using variance inflation factors (VIFs).

Logistic regression was performed in various different subpopulations. We built models to predict hyperbilirubinemia without kernicterus and kernicterus alone and by GA groupings for hyperbilirubinemia without kernicterus (no such analyses were done for the kernicterus subgroup given the small sample size by GA grouping). We also assessed the performance of the models when the population was stratified by hyperbilirubinemia without kernicterus with and without diagnosis before birth discharge diagnosis. We evaluated the performance of the models in both the training and testing subsets using the area under the receiver operating characteristic curve (AUC) and cross‐validated the results. All statistical analyses were performed using the software SAS version 9.4 (SAS Institute, Cary, NC).

Methods and protocols for the study were approved by the Committee for the Protection of Human Subjects within the Health and Human Services Agency of the State of California and the institutional review board of the University of California, San Francisco.

## Results

Of the 1,270,244 infants in the training data set, 234,429 were diagnosed with hyperbilirubinemia without kernicterus, and 26 were diagnosed with kernicterus. Most (86.53%) of those with hyperbilirubinemia without kernicterus were diagnosed before being discharged from the hospital whereas only 7.69% of those with kernicterus were diagnosed before discharge for the birth (**Table** 1). We found that 11 of 13 infant and maternal characteristics as well as 41 of 42 metabolites exhibited crude differences between exposed and unexposed infants with hyperbilirubinemia and no kernicterus (**Table** 2). In infants with kernicterus, two characteristics and 10 metabolites had a *P* < 0.10 (**Table** 2). Lower levels of TSH (OR: 0.79; 95% CI: 0.78–0.79) and higher levels of acylcarnitines C‐16 and C‐18:1 (OR: 1.56; 95% CI: 0.54–0.58 and OR: 1.57; 95% CI: 1.54–1.59, respectively) were associated with particularly increased crude risk of hyperbilirubinemia. Increased phenylalanine (OR: 7.23; 95% CI: 1.86–28.14), valine (OR: 4.17; 95% CI: 1.14–15.17), and C‐3 (OR: 3.50, 95% CI: 1.32–9.27) were associated with particularly increased risk of kernicterus in crude models.

**Table 1:** Maternal and infant characteristics in the training data set by hyperbilirubinemia status^a^

	Hyperbilirubinemia without kernicterus	Kernicterus	No hyperbilirubinemia
	*n* = 234,429	*n* = 26	*n* = 1,035,789
Newborn			
Gestational age			
< 34 weeks	0.21%	0.00%	0.08%
34–36 weeks	6.89%	7.69%	3.26%
≥ 37 weeks	92.90%	92.31%	96.66%
Birthweight (grams), mean (±SD)	3342.16 (484.14)	3338.08 (554.71)	3388.09 (448.06)
Age at newborn screening (hours), mean (±SD)	33.64 (16.29)	32.92 (21.88)	29.99 (13.40)
Sex			
Male	52.58%	80.77%	50.78
Female	47.42%	19.23%	49.22%
Total parenteral nutrition	1.07%	3.85%	0.31%
Feeding type			
Breast milk	53.17%	50.00%	50.74%
Formula	7.74%	0.00%	10.03%
Mixed	39.09%	50.00%	39.23%
Diagnosis before birth discharge	86.53%	7.69%	–
Maternal Race			
White	24.72%	7.69%	26.14%
African American	2.52%	0.00%	3.72%
Hispanic	44.97%	65.38%	51.05%
Asian	20.55%	19.23%	12.61%
Other	7.24%	7.69%	6.49%
Education			
< 12 years	22.61%	26.92%	25.76%
12 years	21.12%	15.38%	23.88%
> 12 years	52.15%	53.85%	46.92%
Unknown	4.12%	3.85%	3.44%
MediCal recipient	42.45%	50.00%	46.11%
Hypertensive	7.36%	15.38%	5.15%
Diabetic	10.11%	26.92%	7.83%
Obese BMI (≥ 30)	20.90%	23.08%	20.52%
Pregnancy infection	1.79%	0.00%	1.74%
Pregnancy smoking	2.52%	3.85%	3.46%

BMI, body mass index.

a *P* < 0.0001 for the χ^2^ test across groups except for pregnancy infection (*P* = 0.2039).

**Table 2:** Crude ORs in training data set

	Hyperbilirubinemia without kernicterus		Kernicterus	
	OR (95% CI)	*P* value	OR (95% CI)	*P* value
Newborn				
Gestational age (weeks)	0.87 (0.87–0.87)	< 0.0001	0.72 (0.56–0.93)	0.0111
Birthweight (grams)	1.00 (1.00–1.00)	< 0.0001	1.00 (0.99–1.00)	0.3374
Age at newborn screening	1.02 (1.02–1.02)	< 0.0001	1.01 (0.98–1.03)	0.6075
Sex				
Male	Reference		Reference	
Female	0.93 (0.92–0.94)	< 0.0001	0.25 (0.09–0.65)	0.0047
Feeding type				
Breast milk	Reference		Reference	
Formula	0.73 (0.72–0.74)	< 0.0001	–	
Mixed	0.94 (0.93–0.95)	< 0.0001	0.95 (0.44–2.06)	0.9616
Maternal Race				
White	Reference		Reference	
African American	0.72 (0.70–0.74)	< 0.0001	–	
Hispanic	0.93 (0.92–0.94)	< 0.0001	2.56 (0.74–8.83)	0.9638
Asian	1.72 (1.70–1.75)	< 0.0001	3.44 (0.82–14.41)	0.9590
Other	1.18 (1.16–1.20)	< 0.0001	4.02 (0.81–19.93)	0.9566
Education				
< 12 years	0.99 (0.98–1.01)	< 0.0001	1.62 (0.47–5.54)	0.8418
12 years	Reference		Reference	
> 12 years	1.27 (1.25–1.28)	< 0.0001	1.78 (0.59–5.41)	0.6243
Unknown	1.37 (1.33–1.40)	< 0.0001	1.73 (0.19–15.49)	0.8487
MediCal recipient	0.86 (0.85–0.87)	< 0.0001	1.00 (0.46–2.17)	0.9969
Hypertensive	1.46 (1.44–1.49)	< 0.0001	2.41 (0.72–8.02)	0.1526
Diabetic	1.31 (1.29–1.33)	< 0.0001	3.51 (1.41–8.75)	0.0069
Obese BMI (≥30)	0.99 (0.98–1.01)	0.2667	0.89 (0.32–2.49)	0.8199
Pregnancy infection	1.02 (0.98–1.05)	0.3181	–	
Pregnancy smoking	0.73 (0.71–0.75)	< 0.0001	–	0.9774
Metabolites				
Enzymes and hormones				
17 Hydroxyprogesterone	0.88 (0.87–0.89)	< 0.0001	2.05 (1.06–3.95)	0.0321
Thyroid stimulating hormone	0.79 (0.78–0.79)	< 0.0001	0.84 (0.47–1.53)	0.5750
Galactose‐1‐phosphate uridyl transferase	1.16 (1.14–1.19)	< 0.0001	1.08 (0.18–6.46)	0.9323
Amino acids				
5‐Oxoproline	1.02 (1.01–1.02)	0.0004	1.31 (0.59–2.91)	0.5140
Alanine	1.03 (1.01–1.04)	0.0007	1.01 (0.28–3.66)	0.9897
Arginine	1.06 (1.05–1.07)	< 0.0001	2.12 (1.13–3.94)	0.0199
Citrulline	0.93 (0.91–0.94)	< 0.0001	1.58 (0.38–6.56)	0.5321
Glycine	1.28 (1.26–1.30)	< 0.0001	3.05 (0.72–12.99)	0.1315
Isoleucine + leucine	1.36 (1.33–1.38)	< 0.0001	1.38 (0.31–6.24)	0.6748
Methionine	1.07 (1.05–1.09)	< 0.0001	3.72 (0.95–14.56)	0.0590
Ornithine	1.09 (1.08–1.11)	< 0.0001	3.47 (1.14–10.55)	0.0284
Phenylalanine	1.37 (1.34–1.40)	< 0.0001	7.23 (1.86–28.14)	0.0043
Proline	1.37 (1.35–1.39)	< 0.0001	0.97 (0.27–3.50)	0.9577
Tyrosine	1.45 (1.43–1.47)	< 0.0001	3.03 (1.09–8.40)	0.0333
Valine	1.22 (1.20–1.24)	< 0.0001	4.17 (1.14–15.17)	0.0306
Carnitines				
Free carnitine	1.01 (1.00–1.02)	0.0698	1.87 (0.77–4.57)	0.1702
C‐2	1.26 (1.24–1.28)	< 0.0001	1.42 (0.42–4.82)	0.5694
C‐3	1.16 (1.14–1.17)	< 0.0001	3.50 (1.32–9.27)	0.0119
C‐3DC	1.41 (1.40–1.43)	< 0.0001	0.60 (0.22–1.63)	0.3170
C‐4	1.16 (1.14–1.17)	< 0.0001	1.20 (0.55–2.67)	0.6613
C‐5	1.12 (1.11–1.13)	< 0.0001	1.09 (0.52–2.27)	0.8245
C‐5:1	1.01 (1.01–1.02)	< 0.0001	0.75 (0.49–1.16)	0.1969
C‐5DC	1.27 (1.26–1.28)	< 0.0001	1.10 (0.47–2.55)	0.8323
C‐5OH	1.06 (1.05–1.07)	< 0.0001	1.37 (0.56–3.30)	0.4903
C‐6	1.05 (1.05–1.06)	< 0.0001	1.75 (0.91–3.36)	0.0939
C‐8	1.11 (1.10–1.12)	< 0.0001	1.66 (0.84–3.27)	0.1463
C‐8:1	1.02 (1.01–1.03)	< 0.0001	1.02 (0.54–1.92)	0.9535
C‐10	1.15 (1.14–1.16)	< 0.0001	1.16 (0.59–2.29)	0.6746
C‐10:1	1.07 (1.06–1.07)	< 0.0001	2.00 (0.94–4.25)	0.0720
C‐12	1.13 (1.12–1.14)	< 0.0001	1.80 (0.84–3.85)	0.1299
C‐12:1	1.10 (1.09–1.11)	< 0.0001	1.47 (0.80–2.73)	0.2182
C‐14	1.46 (1.45–1.48)	< 0.0001	1.71 (0.58–5.40)	0.3316
C‐14:1	1.28 (1.27–1.30)	< 0.0001	1.21 (0.54–2.73)	0.6437
C‐14OH	1.06 (1.05–1.07)	< 0.0001	0.93 (0.58–1.49)	0.7469
C‐16	1.56 (1.54–1.58)	< 0.0001	1.19 (0.36–3.94)	0.7825
C‐16:1	1.33 (1.32–1.35)	< 0.0001	1.02 (0.41–2.55)	0.9604
C‐16OH	1.08 (1.07–1.08)	< 0.0001	1.81 (1.00–3.28)	0.0493
C‐18	1.40 (1.38–1.42)	< 0.0001	1.88 (0.58–6.07)	0.2930
C‐18:1	1.57 (1.54–1.59)	< 0.0001	2.86 (0.82–10.06)	0.1009
C‐18:1OH	1.07 (1.06–1.07)	< 0.0001	0.85 (0.53–1.36)	0.4929
C‐18:2	1.03 (1.02–1.04)	< 0.0001	1.01 (0.47–2.20)	0.9726
C‐18OH	1.06 (1.06–1.07)	< 0.0001	1.21 (0.75–1.96)	0.4345

BMI, body mass index; CI, confidence interval; OR, odds ratio.

Reference for all ORs is no hyperbilirubinemia of any kind.

**Figure 1:**
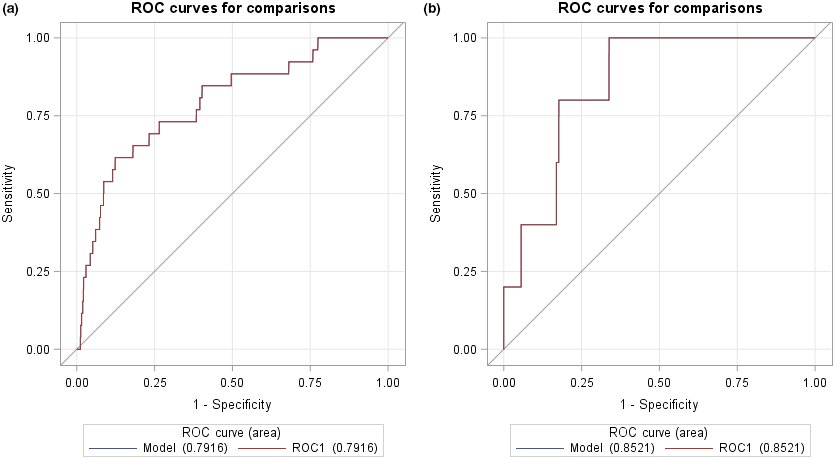
Receiver operating curves (ROCs) for kernicterus in the (**a**) training and (**b**) testing data sets.

In final multivariable models, one infant and maternal characteristics and 31 metabolites were found to be associated with hyperbilirubinemia without kernicterus (**Table** 3). The most strongly associated metabolites were phenylalanine (OR: 1.91; 95% CI: 1.85–1.97), ornithine (OR: 0.76; 95% CI: 0.74–0.77), and isoleucine + leucine (OR: 0.63; 95% CI: 0.61–0.65) with an AUC across all characteristics and metabolites of 0.633 (95% CI: 0.632–0.634) in the training set and 0.633 (05% CI: 0.631–0.636) in the testing set. The C‐3 was associated with increased risk of kernicterus in adjusted models (OR: 3.17; 95% CI: 1.18–8.53) along with GA in weeks (wherein older GA was associated with decreased risk; OR: 0.75; 95% CI: 0.58–0.98) with an AUC of 0.792 (95% CI: 0.699–0.884) in the training set and 0.852; 95% CI: 0.738–0.966 in the testing set (**Figure** 1).

**Table 3:** Multivariable models for hyperbilirubinemia without kernicterus (vs. no hyperbilirubinemia), kernicterus (vs. no hyperbilirubinemia) and kernicterus vs. hyperbilirubinemia without kernicterus

	Hyperbilirubinemia without kernicterus (vs. none)^a^		Kernicterus (vs. none)^b^	
	OR (95% CI)	Standardized estimate (*P* value)	OR (95% CI)	Standardized estimate (*P* value)
Newborn				
Gestational age (weeks)	0.90 (0.90–0.91)	−0.0694 (< 0.0001)	0.75 (0.58–0.98)	−0.1902 (0.0317)
Birthweight (grams)	1.00 (1.00–1.00)	−0.0196 (<0 .0001)	–	–
Age at newborn screening	1.02 (1.02–1.02)	0.1640 (< 0.0001)	1.01 (0.98–1.03)	0.0570 (0.5523)
Sex				
Male	Reference		Reference	
Female	0.95 (0.94–0.96)	−0.0152 (<0.0001)	0.26 (0.10–0.70)	−0.3682 (0.0073)
Feeding type				
Breast milk	Reference		Reference	Reference
Formula	0.84 (0.82–0.85)	−0.0383 (<0.0001)	–	–
Mixed	0.96 (0.95–0.97)	0.0159 (< 0.0001)	0.75 (0.34–1.67)	1.9131 (0.9475)
Maternal Race			–	–
White	Reference		–	–
African American	0.75 (0.72–0.77)	−0.0993 (< 0.0001)	–	–
Hispanic	1.00 (0.99–1.01)	−0.0334 (< 0.0001)	–	–
Asian	1.68 (1.65–1.71)	−0.0334 (< 0.0001)	–	–
Other	1.15 (1.12–1.17)	0.0190 (< 0.0001)	–	–
Education				
< 12 years	1.03 (1.02–1.05)	−0.0199 (<0.0001)	–	–
12 years	Reference		–	–
> 12 years	1.09 (1.08–1.11)	0.00265 (0.2279)	–	–
Unknown	1.24 (1.21–1.27)	0.0343 (< 0.0001)	–	–
MediCal recipient	1.03 (1.02–1.04)	0.00851 (<0.0001)	–	–
Hypertensive	1.34 (1.32–1.37)	0.0372 (< 0.0001)	–	–
Diabetic	1.16 (1.14–1.18)	0.0221 (< 0.0001)	2.95 (1.18–7.42)	0.1607 (0.0213)
Obese BMI (≥30)	–	–	–	–
Pregnancy infection	–	–	–	–
Pregnancy smoking	0.78 (0.76–0.81)	−0.0241 (< 0.0001)	–	–
Metabolites				
Enzymes and hormones				
17 Hydroxyprogesterone	0.91 (0.90–0.92)	−0.0318 (< 0.0001)	–	–
Thyroid stimulating hormone	0.93 (0.92–0.94)	−0.0256 (< 0.0001)	–	–
Galactose‐1‐phosphate uridyl transferase	1.03 (1.00–1.05)	0.00311 (0.0320)	–	–
Amino acids				
5‐Oxoproline	–	–	–	–
Alanine	–	–	–	–
Arginine	1.02 (1.01–1.03)	0.00592 (< 0.0001)	–	–
Citrulline	0.84 (0.82–0.86)	−0.0258 (< 0.0001)	–	–
Glycine	1.25 (1.22–1.28)	0.0307 (< 0.0001)	–	–
Isoleucine + leucine	0.63 (0.61–0.65)	−0.0657 (< 0.0001)	–	–
Methionine	1.10 (1.08–1.12)	0.0143 (< 0.0001)	–	–
Ornithine	0.76 (0.74–0.77)	−0.0492 (< 0.0001)	–	–
Phenylalanine	1.91 (1.85–1.97)	0.0758 (< 0.0001)	–	–
Proline	1.19 (1.16–1.21)	0.0286 (< 0.0001)	–	–
Tyrosine	1.27 (1.25–1.29)	0.0473 (< 0.0001)	–	–
Valine	0.92 (0.90–0.94)	−0.0129 (< 0.0001)	–	–
Carnitines				
Free carnitine	–	–	–	–
C‐2	0.92 (0.90–0.94)	−0.0143 (< 0.0001)	–	–
C‐3	1.15 (1.13–1.17)	0.0298 (< 0.0001)	3.17 (1.18–8.53)	0.2432 (0.0223)
C‐3DC	1.07 (1.05–1.08)	0.0141 (< 0.0001)	–	–
C‐4	1.03 (1.02–1.04)	0.00753 (< 0.0001)	–	–
C‐5	1.01 (1.00–1.02)	0.00339 (0.01845)	–	–
C‐5:1	–	–	–	–
C‐5DC	–	–	–	–
C‐5OH	–	–	–	–
C‐6	0.99 (0.94–1.00)	−0.00440 (0.0012)	–	–
C‐8	1.01 (1.00–1.02)	0.00343 (0.0161)	–	–
C‐8:1	0.95 (0.95–0.96)	−0.0157 (< 0.0001)	–	–
C‐10	0.98 (0.97–0.99)	−0.00781 (< 0.0001)	–	–
C‐10:1	0.99 (0.98–0.99)	−0.00493 (0.0003)	–	–
C‐12	0.95 (0.94–0.97)	−0.0140 (< 0.0001)	–	–
C‐12:1	1.01 (1.00–1.02)	0.00340 (0.0471)	–	–
C‐14	1.04 (1.02–1.06)	0.00831 (< 0.0001)	–	–
C‐14:1	1.07 (1.06–1.09)	0.0189 (< 0.0001)	–	–
C‐14OH	–	–	–	–
C‐16	1.09 (1.06–1.13)	0.0159 (< 0.0001)	–	–
C‐16:1	1.15 (1.13–1.17)	0.0322 (< 0.0001)	–	–
C‐16OH	1.02 (1.01–1.02)	0.00619 (< 0.0001)	–	–
C‐18	1.05 (1.02–1.07)	0.00825 (< 0.0001)	–	–
C‐18:1	–	–	–	–
C‐18:1OH	1.01 (1.01–1.02)	0.00493 (0.0003)	–	–
C‐18:2	0.90 (0.89–0.91)	−0.0307 (< 0.0001)	–	–
C‐18OH	–	–	–	–

AUC, area under the receiver operating characteristic curve; BMI, body mass index; CI, confidence interval; OR, odds ratio.

^a^AUC: 0.6330 (0.6318–0.6343); ^b^AUC: 0.7916 (0.6992–0.8840).

When stratified by GA, several patterns remained consistent across GA groups for hyperbilirubinemia without kernicterus. TSH and C‐18:2 were found to be decreased (OR: 0.93; 95% CI: 0.92–0.93; OR: 0.96; 95% CI: 0.93–0.99; OR: 0.82; 95% CI: 0.70–0.96, > 36, 34–36 and < 32 weeks, respectively, for TSH, and OR: 0.89; 95% CI: 0.88–0.90; OR: 0.90; 95% CI: 0.86–0.95; OR: 0.69; 95% CI: 0.53–0.90, 36, 34–36, and <32 weeks, respectively, for C‐18:2) while tyrosine and C‐3 were increased in infants across groupings (OR: 1.29; 1.27–1.35; OR: 1.18; 95% CI: 1.12–1.25; OR: 1.37; 95% CI: 1.07–1.75, >36, 34–36 and <32 weeks, respectively, for tyrosine and OR: 1.12; 95% CI: 1.10–1.14; OR: 1.34; 95% CI: 1.25–1.43; OR: 1.96; 95% CI: 1.42–2.69, >36, 34–36, and < 32 weeks, respectively, for C‐3; **Table** 4).

**Table 4:** Multivariable models by gestational age (> 36 weeks, 34–36 weeks, and < 34 weeks) for hyperbilirubinemia without kernicterus

	> 36 weeks GA^a^		34–36 weeks GA^b^		< 34 weeks GA^c^	
	OR (95% CI)	Standardized estimate (*P* value)	OR (95% CI)	Standardized estimate (*P* value)	OR (95% CI)	Standardized estimate (*P* value)
Newborn			–			
GA (weeks)	0.95 (0.94–0.95)	−0.0329 (< 0.0001)	0.81 (0.78–0.83)	−0.0735 (< 0.0001)		
Birthweight (grams)	1.00 (1.00–1.00)	−0.00958 (< 0.0001)	1.00 (1.00–1.00)	−0.0697 (< 0.0001)	1.00 (1.00–1.00)	−0.1704 (< 0.0001)
Age at newborn screening	1.02 (1.02–1.02)	0.1551 (< 0.0001)	1.02 (1.02–1.02)	0.2371 (< 0.0001)	1.01 (1.01–1.02)	0.1750 (0.0002)
Sex						
Male	Reference		–		–	
Female	0.95 (0.94–0.96)	−0.0155 (< 0.0001)	–		–	
Feeding type						
Breast milk	Reference		Reference		Reference	
Formula	0.83 (0.81–0.84)	−0.0396 (< 0.0001)	0.77 (0.72–0.83)	−0.0516 (< 0.0001)	1.47 (0.98–2.22)	0.0370 (0.3149)
Mixed	0.95 (0.94–0.96)	0.0143 (< 0.0001)	0.91 (0.87–0.95)	0.0103 (0.1466)	1.57 (1.10–2.23)	0.0722 (0.0480)
Maternal Race						
White	Reference		Reference		Reference	
African American	0.76 (0.73–0.78)	−0.0993 (< 0.0001)	0.57 (0.51–0.64)	−0.1160 (< 0.0001)	0.38 (0.21–0.69)	−0.1456 (0.0089)
Hispanic	1.02 (1.00–1.03)	−0.0333 (< 0.0001)	0.80 (0.76–0.85)	−0.0432 (< 0.0001)	0.75 (0.52–1.08)	0.0479 (0.2770)
Asian	1.73 (1.70–1.76)	0.1582 (< 0.0001)	1.26 (1.18–1.34)	0.1173 (< 0.0001)	0.88 (0.53–1.44)	0.0714 (0.1234)
Other	1.16 (1.14–1.19)	0.0185 (< 0.0001)	0.93 (0.86–1.02)	0.0160 (0.0904)	0.53 (0.30–0.93)	−0.0557 (0.2692)
Education						
< 12 years	1.04 (1.03–1.06)	−0.0169 (< 0.0001)	0.97 (0.91–1.02)	−0.0385 (< 0.0001)	–	
12 years	Reference		Reference		–	
> 12 years	1.09 (1.08–1.11)	0.000047 (0.9834)	1.11 (1.05–1.17)	0.0176 (0.0562)	–	
Unknown	1.24 (1.21–1.27)	0.0343 (< 0.0001)	1.20 (1.08–1.34)	0.0320 (0.0027)	–	
MediCal recipient	1.05 (1.04–1.06)	0.0134 (< 0.0001)	0.84 (0.80–0.88)	−0.0486 (< 0.0001)	0.61 (0.47–0.79)	−0.1314 (0.0002)
Hypertensive	1.33 (1.30–1.35)	0.0344 (< 0.0001)	1.35 (1.27–1.42)	0.0577 (< 0.0001)	–	
Diabetic	1.16 (1.14–1.18)	0.0219 (< 0.0001)	1.23 (1.16–1.30)	0.0375 (< 0.0001)	–	
Obese BMI (≥ 30)	–				–	
Pregnancy infection	–				–	
Pregnancy Smoking	0.79 (0.77–0.81)	−0.0231 (< 0.0001)	0.69 (0.62–0.76)	−0.0429 (< 0.0001)	–	
Metabolites						
Enzymes and hormones						
17 hydroxyprogesterone	0.90 (0.89–0.90)	−0.0376 (< 0.0001)	1.14 (1.11–1.18)	0.0494 (< 0.0001)	–	
Thyroid stimulating hormone	0.93 (0.92–0.93)	−0.0265 (< 0.0001)	0.96 (0.93–0.99)	−0.0155 (0.0187)	0.82 (0.70–0.96)	−0.0988 (0.0146)
Galactose‐1‐phosphate uridyl transferase	1.04 (1.02–1.07)	0.00498 (0.0007)	–		–	
Amino acids					–	
5‐Oxoproline	–		–		–	
Alanine	–		–		–	
Arginine	–		1.05 (1.01–1.08)	0.0171 (0.0051)	–	
Citrulline	0.84 (0.82–0.86)	−0.0258 (< 0.0001)	0.83 (0.77–0.90)	−0.0289 (< 0.0001)	–	
Glycine	1.27 (1.24–1.30)	0.0331 (< 0.0001)	–		–	
Isoleucine + leucine	0.61 (0.59–0.63)	−0.0688 (< 0.0001)	0.77 (0.69–0.86)	−0.0391 (< 0.0001)	–	
Methionine	1.12 (1.09–1.14)	0.0169 (< 0.0001)	–		–	
Ornithine	0.77 (0.75–0.78)	−0.0465 (< 0.0001)	0.72 (0.67–0.78)	−0.0617 (< 0.0001)	–	
Phenylalanine	1.83 (1.78–1.89)	0.0707 (< 0.0001)	2.49 (2.21–2.81)	0.1136 (< 0.0001)	–	
Proline	1.20 (1.18–1.23)	0.0303 (< 0.0001)	–		–	
Tyrosine	1.29 (1.27–1.35)	0.0490 (< 0.0001)	1.18 (1.12–1.25)	0.0382 (< 0.0001)	1.37 (1.07–1.75)	0.0862 (0.0130)
Valine	0.92 (0.90–0.94)	−0.0136 (< 0.0001)	–		–	
Carnitines						
Free carnitine	–		0.81 (0.74–0.88)	−0.0520 (< 0.0001)	–	
C‐2	0.93 (0.91–0.95)	−0.0132 (< 0.0001)	1.13 (1.02–1.27)	0.0223 (0.0259)	–	
C‐3	1.12 (1.10–1.14)	0.0241 (< 0.0001)	1.34 (1.25–1.43)	0.0631 (< 0.0001)	1.96 (1.42–2.69)	0.1662 (< 0.0001)
C‐3DC	1.03 (1.01–1.05)	0.00583 (0.0047)	0.88 (0.82–0.94)	−0.0288 (0.0003)	–	
C‐4	1.03 (1.02–1.04)	0.00749 (< 0.0001)	–		–	
C‐5	–		1.06 (1.02–1.11)	0.0173 (0.0060)	–	
C‐5:1	–		–		–	
C‐5DC	1.06 (1.04–1.08)	0.0157 (< 0.0001)	–		–	
C‐5OH	–		–		1.34 (1.00–1.79)	0.0783 (0.0476)
C‐6	0.99 (0.98–0.99)	−0.00568 (< 0.0001)	–		–	
C‐8	–		–		–	
C‐8:1	–		0.95 (0.92–0.98)	−0.0168 (0.0036)	–	
C‐10	0.98 (0.97–0.99)	−0.00627 (< 0.0001)	–		–	
C‐10:1	0.98 (0.98–0.99)	−0.00704 (< 0.0001)	–		–	
C‐12	–		0.82 (0.79–0.86)	−0.0615 (< 0.0001)	–	
C‐12:1	1.02 (1.01–1.03)	0.00696 (< 0.0001)	–		–	
C‐14	1.06 (1.04–1.08)	0.0122 (< 0.0001)	1.08 (1.01–1.16)	0.0166 (0.0361)	–	
C‐14:1	–		–		–	
C‐14OH	–		–		–	
C‐16	1.11 (1.12–1.16)	0.0180 (< 0.0001)	–		–	
C‐16:1	1.14 (1.12–1.16)	0.0309 (< 0.0001)	1.28 (1.20–1.36)	0.0632 (< 0.0001)	–	
C‐16OH	1.02 (1.01–1.02)	0.00719 (< 0.0001)	–		–	
C‐18	1.04 (1.02–1.07)	0.00809 (< 0.0001)	1.13 (1.05–1.22)	0.0231 (0.0014)	–	
C‐18:1	–				–	
C‐18:1OH	1.01 (1.00–1.02)	0.00398 (0.0043)	1.03 (1.00–1.05)	0.0122 (0.0332)	–	
C‐18:2	0.89 (0.88–0.90)	−0.0331 (< 0.0001)	0.90 (0.86–0.95)	−0.0298 (< 0.0001)	0.69 (0.53–0.90)	−0.1205 (0.0053)
C‐18OH	–		–			

AUC, area under the receiver operating characteristic curve; BMI, body mass index; CI, confidence interval; GA, gestational age; OR, odds ratio.

^a^AUC: 0.6263 (95% CI: 0.6250–0.6276); ^b^AUC: 0.6665 (95% CI: 0.6615–0.6715); ^c^AUC: 0.7109 (95% CI: 0.6828–0.7389).

Among infants of GA > 36 weeks, 11 characteristics and a total of 28 metabolites were associated with hyperbilirubinemia without kernicterus (**Table** 4; AUC: 0.626; 95% CI: 0.625–0.628) in the training set and 0.627; 95% CI: 0.625–0.629 in the testing set). Among infants of GA 34–36 weeks, 10 characteristics and 20 metabolites showed associations with hyperbilirubinemia without kernicterus (**Table** 4; AUC: 0.667; 95% CI: 0.662–0.672 in the training set and 0.670; 95% CI: 0.661–0.679 in the testing set). In infants born at < 34 weeks gestation, five characteristics and five metabolites were associated with hyperbilirubinemia (AUC: 0.711; 95% CI: 0.683–0.739 in the training set and AUC: 0.703; 95% CI: 0.654–0.753 in the testing set).

## Discussion

In this study, we observed novel associations between hyperbilirubinemia with and without kernicterus and newborn metabolic profile. Most notably we found consistent relationships between hyperbilirubinemia without kernicterus and lower TSH, higher tyrosine, higher C‐3, and lower C‐18:2 across GA groupings. Higher C‐3 was also found to be associated with kernicterus.

Although it is known that preterm infants are metabolically distinct from their term counterparts,^15^, ^20^ this study demonstrates that even when stratified according to GA, infants with hyperbilirubinemia with and without kernicterus are metabolically distinct from infants without hyperbilirubinemia.

With respect to infant and maternal characteristics, our observed association between maternal smoking during pregnancy and reduced risk of hyperbilirubinemia has been documented elsewhere.^21^ It is thought that these differences may result from compounds in cigarettes that may induce microsomal enzyme systems, which increases the metabolism of bilirubin.^21^ It should be noted that in the present study, smoking was not significantly associated with hyperbilirubinemia in the < 34‐week GA group. This pattern may reflect the fact that low birthweight infants are less susceptible to hepatic enzyme induction.^21^

This study also confirmed the importance of Asian race, male sex, low birthweight, young GA, maternal hypertension, maternal diabetes, and breastfeeding in observed patterns of risk.^2^, ^3^, ^9^, ^22^ Interestingly, among infants of < 34 weeks GA, higher GA was associated with an increased risk of hyperbilirubinemia. One possible explanation for this finding may be that for extremely premature infants, practitioners administer phototherapy treatment before these infants reach a TSB level that would be recorded with an *ICD‐9* code.

The most consistent finding in the present study was the association between increased C‐3 across hyperbilirubinemia with and without kernicterus groupings (overall and within GA groupings). A previous study by Zhao *et al*.^23^ considered a possible relationship between C‐3 and hyperbilirubinemia, and found that C‐3 tended to be lower in infants with hyperbilirubinemia than their healthy peers and did not examine kernicterus specifically. Although that there are differences between their study and ours, it is of interest that we suspect that this difference may be explained by the small case size in their study (*n* = 32). Although at present it is unknown whether these patterns might point to potentially relevant etiologic pathways, of note is the fact that high C‐3 levels are closely tied to methylmalonic academia and metabolic acidosis.^24^ As such, future research focused on what link C‐3 may have to hyperbilirubinemia and to kernicterus in particular is suggested by this work.

Other consistent patterns observed across GAs among those with hyperbilirubinemia without kernicterus included decreased levels of TSH, increased levels of tyrosine, and decreased levels of C‐18:2. Although it is unclear what may underlie the relationship between hyperbilirubinemia without kernicterus and TSH, others have suggested that lower levels of TSH may suggest thyroid derangements.^25^, ^26^ Tyrosinemia, characterized by increased levels of tyrosine, has also been shown to be related to hyperbilirubinemia, which might be caused transiently by immature liver enzymes.^27^ It may be that infants with hyperbilirubinemia without kernicterus have less fully developed livers than their nonhyperbilirubinemic counterparts. Our finding of consistently lower levels of C‐18:2 across GA groupings in those with hyperbilirubinemia without kernicterus is particularly interesting given well‐established importance of C‐18:2 (also known as linoleic acid) in maintaining good newborn health – particularly in premature infants.^28^

Other findings from the present study may have important pathophysiologic implications. In infants ≥ 34 weeks GA, ornithine was significantly lower in infants with hyperbilirubinemia without kernicterus. Ornithine plays an important role in the urea cycle, which primarily occurs in hepatic cells.^29^, ^30^ Other potentially relevant etiologic underpinnings are suggested by the patterns we observed for leucine and free carnitine. Elevated levels of leucine and isoleucine are consistent with abnormal metabolism of amino acids among infants with hyperbilirubinemia, and have been observed previously in term infants.^1^ However, in the present study, decreased levels of leucine were seen in infants ≥ 34 weeks GA. Low levels of isoleucine and leucine are associated with malnutrition.^31^ Insufficient intake of breast milk during the early neonatal period is a known cause of hyperbilirubinemia.^2^, ^3^, ^9^ Breastfeeding‐related hyperbilirubinemia may be one explanation for the decreased levels of leucine among cases in the two larger GA cohorts. This theory is further supported by the fact that decreased free carnitine levels, seen in the 34–36‐week cohort, have also been observed in malnourished children.^31^, ^32^ Lower free carnitine levels are also associated with decrease synthesis of albumin,^32^, ^33^ which binds unconjugated bilirubin so that it can be excreted.^2^

The findings from this study point to the utility of considering newborn metabolic function at birth in evaluating important risks and etiologic underpinnings of hyperbilirubinemia. Strengths of the study include the use of a diverse and large population‐based sample of newborns, meaning that these models and the underpinnings they points to may be generalizable to many different populations. To our knowledge, this is the first investigation of hyperbilirubinemia to use routinely collected metabolic data.

A key limitation of the study is the lack of information about TSB levels that would have allowed us to assess the severity of hyperbilirubinemia. The lack of these data is likely a primary reason that the models in this study had lower predictive value compared with existing models that include TSB levels.^12^, ^13^, ^14^ We were, however, able to pinpoint biomarkers other than bilirubin that are associated with hyperbilirubinemia and to predict hyperbilirubinemia across a greater range of GAs and ethnicities than any existing models. The addition of change in TSB levels between birth and time of diagnosis as a variable in this study's models would likely improve their predictive performance. Another limitation of our measure for hyperbilirubinemia is that practitioners may use different criteria when determining whether or not to include the *ICD‐9* code for hyperbilirubinemia in hospital discharge records. Some clinicians may use the code for any infant who appears jaundiced, whereas some may only include the code if an infant receives phototherapy. It should also be noted that, in the current study, we chose to include all babies diagnosed with hyperbilirubinemia within the first year of life, although 99.9% of diagnoses were within the first 30 days. Although we believe this was the best approach for addressing all instances of hyperbilirubinemia, we were not well‐powered to examine prediction after 30 days. Finally, we note that the full model and the GA 34–36‐week model were not well‐calibrated under the Hosmer‐Lemeshow Goodness of Fit test. Although the numbers of observed and expected events were closely predicted by the model, the Hosmer‐Lemeshow tests indicated the differences were significant (GA 22–41 weeks *P* < 0.001; GA 34–36 weeks *P* = 0.003). This result may be a consequence of the extremely large data set, rather than evidence that the model is truly poorly calibrated.^34^ The model built with the smallest sample size, the GA < 34 week model, was well‐calibrated (*P *= 0.18).

Metabolites from NBSs were shown to be strongly associated with neonatal hyperbilirubinemia both before and after adjustment for other infant and maternal characteristics. Routinely collected newborn metabolic data point to potentially important pathways involved in hyperbilirubinemia and may be useful to consider in future studies focused on explaining and predicting patterns and on potential interventions aimed at prevention.

## Funding

This work was funded by the Bill and Melinda Gates Foundation (Grant ID: OPP1141549)

## Conflict of Interest

The authors declared no competing interests for this work.

## Author Contributions

M.M. wrote the manuscript. S.O., R.B., K.R., and L.J.P. designed the research. M.M. performed the research. J.W., M.S.M., and E.R. contributed new reagents/analytical tools.
